# *In vivo* two-photon-excited cellular fluorescence of melanin, NAD(P)H, and keratin enables an accurate differential diagnosis of seborrheic keratosis and pigmented cutaneous melanoma

**DOI:** 10.1117/1.JBO.26.7.075002

**Published:** 2021-07-14

**Authors:** Łukasz Szyc, Constantin Scharlach, Holger Haenssle, Christine Fink

**Affiliations:** a Magnosco GmbH, Berlin, Germany; b University of Heidelberg, Department of Dermatology, Heidelberg, Germany

**Keywords:** malignant melanoma, seborrheic keratosis, fluorescence, melanin

## Abstract

**Significance:** Seborrheic keratoses (SKs) are harmless pigmented skin lesions (PSLs) that may be confused clinically not only with other benign conditions but also with cutaneous melanoma (CM). As SKs are one of the most common neoplasms in adults, the importance of their correct diagnosis is high. Misclassifying SK as malignant is not rare and leads to a high number of unnecessary biopsies. On the other hand, misdiagnosing CM as SK may have a large impact on prognosis or therapy.

**Aim:** In the non-invasive technique of dermatofluoroscopy, the fluorophores in melanocytes and keratinocytes are excited *in vivo* with nanosecond laser pulses and the resulting spectrally resolved, melanin-dominated fluorescence signals are used to differentiate between pigmented benign lesions and CM.

**Approach:** In this single-center, non-interventional study, 33 PSLs of 20 patients were scanned with dermatofluoroscopy *in vivo*. For all included cases, dermatofluoroscopic signals were compared to pathology classification.

**Results:** The characteristic spectral features of SK were identified, where the signals are dominated by keratin, NAD(P)H, and melanin. The fluorescence spectra of SKs differed substantially from those of CM: a characteristic spectrum of SK has been identified in 27 of 28 SKs.

**Conclusions:** The high-accuracy differential diagnosis between CM and SK is possible with dermatofluoroscopy.

## 1 Introduction

Early cutaneous melanoma (CM) detection continues to be a crucial factor for patient survival since melanoma still is the deadliest form of skin cancer. To date, the clinical diagnosis remains largely based upon visual inspection and dermoscopy. However, assisting diagnostic technologies for early diagnosis will be of increasing importance in the future.^1^

Techniques that use laser light to generate images with (sub)-cellular resolution at various depths, such as confocal reflectance microscopy^2^ and multiphoton tomography,^3^ demonstrate good diagnostic accuracy. However, extensive training is required to correctly interpret the images, and the final judgment may be subjective.

In contrast, dermatofluoroscopy does not produce depth-resolved images but instead acquires fluorescence spectra from multiple tissue layers simultaneously, with a dichotomous objective output to rule out or confirm the diagnosis of CM.^4^ Dermatofluoroscopy uses two phenomena from non-linear laser spectroscopy: (a) simultaneous and stepwise two-photon-excited fluorescence and (b) frequency doubling of laser light in microcrystalline media. Under conventional excitation conditions, ultra-weak melanin fluorescence is surpassed by autofluorescence of other endogenous fluorophores of the skin such as NAD(P)H coenzymes or flavins. Due to stepwise two-photon absorption, melanin can be excited selectively and its fluorescence becomes detectable, with only little contribution of skin fluorophores of greater quantum yield. Previous studies on freshly excised or paraffin-embedded tissue samples (*ex vivo*) have shown that the melanin-dominated fluorescence spectra after stepwise two-photon excitation differ between CM and melanocytic nevi.^4^^–^^7^ However, this promising novel technology showed some limitations. In a first prospective clinical study, a certain proportion of harmless pigmented lesions were inaccurately classified as being malignant, resulting in a moderate (45%) specificity of the method for in vivo detection of CM. Especially, the differentiation between seborrheic keratosis (SK) and CM turned out to be a major challenge for the tested dermatofluoroscope, where only 6 of 17 SKs (35%) were correctly classified as non-malignant using a shallow neural network-based algorithm.^8^

In a subsequent postmarket clinical follow-up (PMCF) study, an algorithm was trained, where 214 PSLs were labeled according to the diagnosis of three experienced pathologists. The use of a support vector machine (SVM) classifier for the analysis of fluorescence signals resulted in improvement of the diagnostic accuracy of dermatofluoroscopy in melanoma diagnosis: the sensitivity was estimated to be 91.7% with the corresponding specificity of 83.0%.^9^ With this new machine learning algorithm, all four SKs in the independent validation set (165 PSLs) of PMCF study were correctly classified as non-malignant (100%).

SKs are very common benign neoplasms in adults^10^ and their misclassification in the differential diagnosis of CM is a known problem,^11^ also in other emerging technologies, e.g., in electrical impedance spectroscopy.^12^ This diagnostic problem is aggravated by the fact that heavily pigmented SKs can also clinically mimic CM. On the other hand, CM of a more acanthotic and verrucous phenotype may be misinterpreted as pigmented SK.^13^^,^^14^ These clinical and technical difficulties in differentiating CM from SK may lead to inappropriate management and delayed melanoma diagnosis as well as to a high number of unnecessary biopsies. Therefore, there is a relevant clinical need to identify a characteristic fluorescence spectrum for SKs.

The main objective of this retrospective study was to investigate patterns of the melanin-dominated fluorescence spectra in SKs and to confirm the high accuracy of classifying SKs as non-malignant lesions using the CE-certified SVM algorithm on higher number of samples compared to the PMCF study.

## 2 Method

**Fig. 1:**
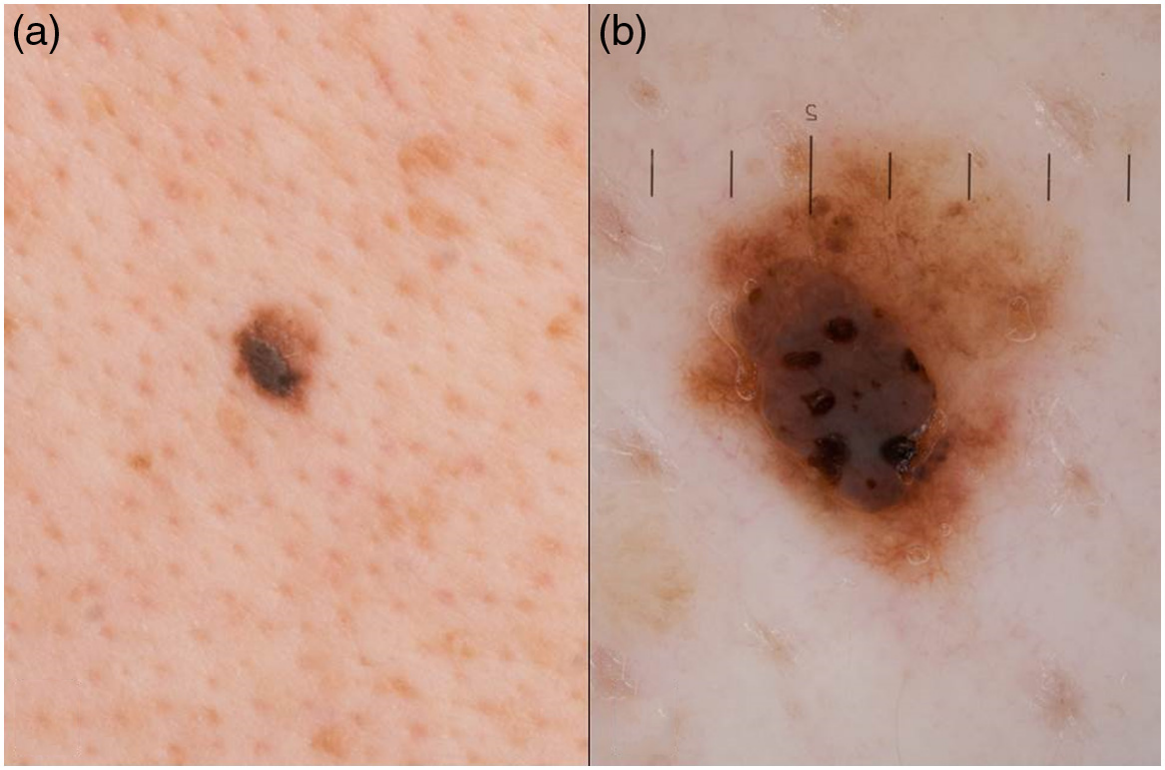
Representative pigmented lesion of this study displayed as (a) overview and (b) dermoscopic image. Histopathology confirmed the clinical diagnosis of a SK and ruled out CM.

This retrospective, non-interventional, single-center analysis of dermatofluoroscopic measurements of 33 PSL was performed in the Department of Dermatology, University of Heidelberg. Aim of this study was to analyze specific fluorescence signals and patterns in pigmented SKs in contrast to melanoma by means of dermatofluoroscopy. All PSLs were selected by trained dermatologists. Only conspicuous skin lesions were selected that showed the simultaneous occurrence of clinical and dermoscopic characteristics of SKs and of a melanocytic entity, such as CM. Since the possible presence of a CM could not be ruled out, all lesions were excised and examined histologically. A representative lesion of this study is shown in Fig. 1.

Within this study, 20 patients of Caucasian skin phenotype (Fitzpatrick skin types 2 and 3) with 33 PSLs were investigated by means of DermaFC in accordance to the intended use of the device. To further investigate the obtained fluorescence signals, comparative measurements on normally pigmented skin of the forearm and the hyperkeratotic skin of fingers and toes of one patient (Fitzpatrick skin type 2) as well as measurements of finger nail were conducted. A phantom sample containing NADH was prepared and measured to obtain pure NADH fluorescence spectra. 0.5 wt. % NADH (Merck, Germany) and 1.2 wt. % ${\mathrm{Al}}_{2}O_{3}$ nanopowder (<50  nm particle size, Merck, Germany) were mixed into a silicone matrix that cures at room temperature (Elastosil RT 604, Wacker, Germany) and allowed to harden under nitrogen atmosphere for 24 h. The fluorescence signals and patterns were compared to data derived from measurements of CM of the PMCF study.^9^ The dermatofluoroscopic scans were performed by the CE-marked class IIa medical device DermaFC (Magnosco GmbH, Berlin, Germany). The laser light is focused in the bottom layers of the epidermis (100±10  μm deep in the skin) with a lateral extension of ∼35  μm. The numerical aperture of the objective lens equals NA=0.28, resulting in a relatively large depth of focus of ∼5  μm. Consequently, the collected signals are integrated over a larger volume of the photoexcited tissue compared to confocal microscopy.

As discussed in detail elsewhere,^9^ dermatofluoroscopy is a score-based method, in which the collected spectra are classified by a machine-learning algorithm. Based on the analysis of this spectrally resolved signals, a score is generated which classifies the lesion as benign or malignant based on a defined threshold. From a score of >28, excision was recommended, the value of the score scales linearly with the number of spectra classified as malignant. The cut-off value of 28 was defined in a PMCF study.^9^ The algorithm used in this study to calculate the score was applied as implemented in the CE-certified medical device DermaFC without any modifications. Depending on the size of the lesion, the measurement took about 4 to 15 min. For all included cases, a clear-cut final diagnosis by surgical excision followed by histopathological analysis was present, and dermatofluoroscopic signals were correlated with histopathological results. The interpretation of the results and the diagnosis were done exclusively by trained dermatologists. The study was conducted in accordance with the declaration of Helsinki principles, and ethical approval was provided by the local ethics committee of the medical faculty of the University of Heidelberg (protocol code: S-020; date of approval: January 15, 2018). Informed consent was obtained from all subjects involved in the study.

## 3 Results

Twenty patients of Caucasian skin phenotype (Fitzpatrick skin types 2 and 3) with a total of 33 PSLs that were clinically diagnosed as SK mimicking CM were included in this study. The average patient age was 68.5 years. Histopathology was used as the reference diagnosis in all lesions. Histopathology confirmed SK in 28 of 33 lesions, whereas the remaining five lesions were diagnosed as benign nevi. Prior to excision, all PSLs were investigated by means of dermatofluoroscopy with the CE-certified medical device DermaFC. Out of 33 lesions, 32 were correctly classified as benign (mean score: 14.6 [95% confidence interval (CI) 12.7 to 16.5], cut-off for malignancy: score >28), which corresponds to a diagnostic accuracy of 97.0% in diagnosing SKs and nevi as non-melanoma with dermatofluoroscopy. All five nevi, clinically classified as SK mimicking malignant melanoma by visual inspection, were correctly diagnosed as benign (mean score: 16.3 [95% CI 11.9 to 20.7]). One lesion, with a score of 33, was erroneously classified as melanoma by dermatofluoroscopy (false positive, histopathology: SK). When including only the 28 histopathologically confirmed SKs into the analysis, we found a correct diagnosis by dermatofluoroscopy in 96.4% of lesions (mean score: 14.4 [95% CI 12.3 to 16.4]) (Table 1). In comparison, in a previous prospective study, the old, neural network-based algorithm classified only 6 of 17 SKs (35%) correctly as non-malignant.^8^

**Table 1:** DermaFC results (score) in comparison to histopathology (gold standard).

Diagnosis according to histopathology	Correct diagnosis by dermatofluoroscopy number, (%), mean score	Incorrect diagnosis by dermatofluoroscopy number, (%), mean score
SK (n=28)	27 (96.4%), 14.4	1 (3.6%), 33.0
Melanocytic nevus (n=5)	5 (100%), 16.3	0 (0.0%), —
CM (n=24)^a^	*22 (91.7%), 37.5*	*2 (8.3%), 18.5*

a Data derived from measurements of CM of the PMCF.^9^

**Fig. 2:**
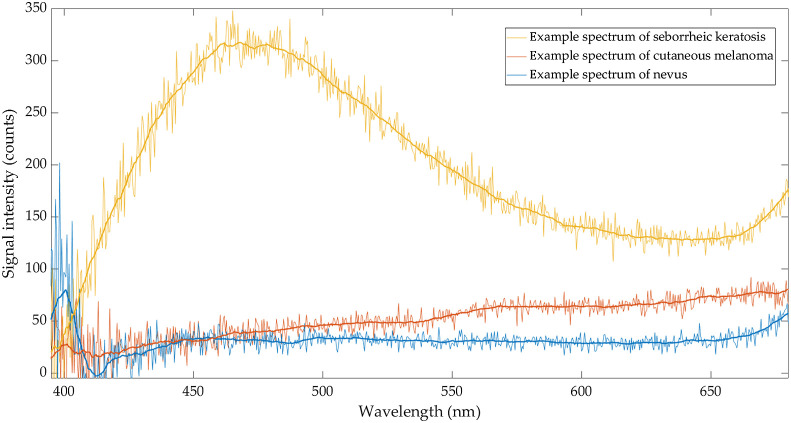
Representative spectra of a SK, CM, and a nevus. Note relatively high intensity of collagen second harmonic at 400 nm in melanocytic nevus (nevus type spectrum, blue) and low-signal intensity of other skin fluorophores. In contrast, a low SHG-signal and very high intensities at 450 to 490 nm, peaking at 475 nm are characteristic for SK (SK type spectrum, yellow). In melanoma (CM type spectrum, red), the intensity of second harmonic at 400 nm is lower than in nevus, whereas the fluorescence intensity in the range between 550 and 650 nm is higher.

In order to identify potential spectroscopic patterns of SK, the spectra were analyzed for common features. The spectra were smoothed using moving average filter of span=40. Typical characteristics could be determined for nearly all evaluated SKs (27 of 28, Table 2). A typical spectrum of SK (“SK type spectrum”) exhibits low or very low signal at 400 nm. This is in stark contrast to nevi, where an intense second harmonic signal indicates the intact microcrystalline structure of collagen in the dermis and/or absence of acanthosis (“nevus type spectrum”). As the most distinctive difference, a very high signal intensity in the spectral range of 450 to 490 nm was observed in SK, which is up to 10 times higher than typical fluorescence intensities of nevi or melanoma. The fluorescence spectrum characteristic for CM (“CM type spectrum”) shows a monotonously increasing intensity toward longer wavelength in the range between 430 and 650 nm (independent of its subtype, including choroidal melanoma). A concomitant decrease of signal intensity at 400 nm suggests a thicker or an invasive melanoma (Fig. 2).

**Table 2:** DermaFC results (spectral characteristics) in comparison to histopathology (gold standard).

Diagnosis according to histopathology	Number of lesions with given spectral characteristics		
	SK type	CM type	Nevus type
SK (n=28)	27	1	0
Melanocytic nevus (n=5)	1	0	4
CM (n=24)^a^	*0*	*22*	*2*

a Data derived from measurements of CM of the PMCF study.^9^

Yet, in dermatofluoroscopy, not all observed spectra exhibit characteristics typical for a distinct skin condition, i.e., a certain fraction of spectra contains no specific information required for diagnosis. Moreover, usually around 5% of measured spectra are removed prior the classification as the signal-to-noise ratio in the defined spectral range is too low for a reliable analysis.

To further investigate the origin of the signal maximum at around 475 nm, comparative measurements on normally pigmented hyperkeratotic skin of toe (plantar side) and finger (palmar side) were analyzed. In the case of hyperkeratotic skin of palms and soles, the laser light is focused in upper parts of the epidermis, and the corresponding signals should be dominated by fluorescence of terminally differentiated keratinocytes (corneocytes) of cornified and translucent layers (stratum corneum and stratum lucidum, respectively). On the contrary, for non-hyperkeratotic skin areas, the excitation light is focused in the basal layer of the epidermis (stratum basale) and the corresponding dermatofluoroscopic signals should be dominated by fluorescence of metabolizing cells such as melanocytes and basal keratinocytes.

**Fig. 3:**
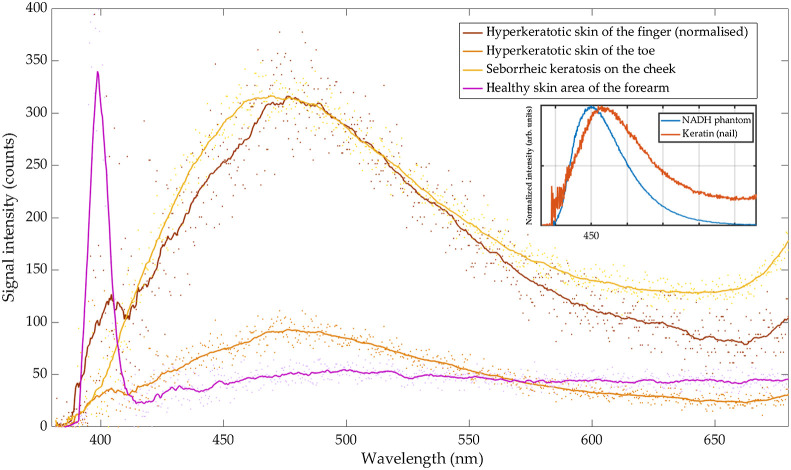
Smoothed (solid line) and raw (dots) example dermatofluoroscopic spectra of: hyperkeratotic skin of the finger (brown, normalized to SK’s maximum intensity), hyperkeratotic skin of the toe (orange), SK (yellow), and healthy skin area of the forearm (purple). Inset: the pure fluorescence spectra of: keratin of human’s nail (red line) and NADH coenzyme in a silicone phantom (blue line).

In comparison to the fluorescence maximum of pigmented SK’s, a slightly red-shifted peak at around 460 to 490 nm was detected for both: hyperkeratotic toe and hyperkeratotic finger. A corresponding measurement of a normally pigmented skin area of a forearm (ventral, thin stratum corneum) illustrates the difference to thickened stratum corneum: the low-intensity fluorescence signal in the spectral range 380 to 680 nm is accompanied by an intense peak at around 400 nm as a result of the second harmonic generation in the collagen of the intact dermis (Fig. 3).

In addition to the measurements of skin and skin tumors, the dermatofluoroscopic spectra of “pure” keratin (inset in Fig. 3, red line) as well as “pure” NADH (inset in Fig. 3, blue line) were measured. The signal of keratin from human nail peaked at around 470 nm, whereas the fluorescence of NADH coenzyme in a custom-made phantom peaked at 450 nm.

**Fig. 4:**
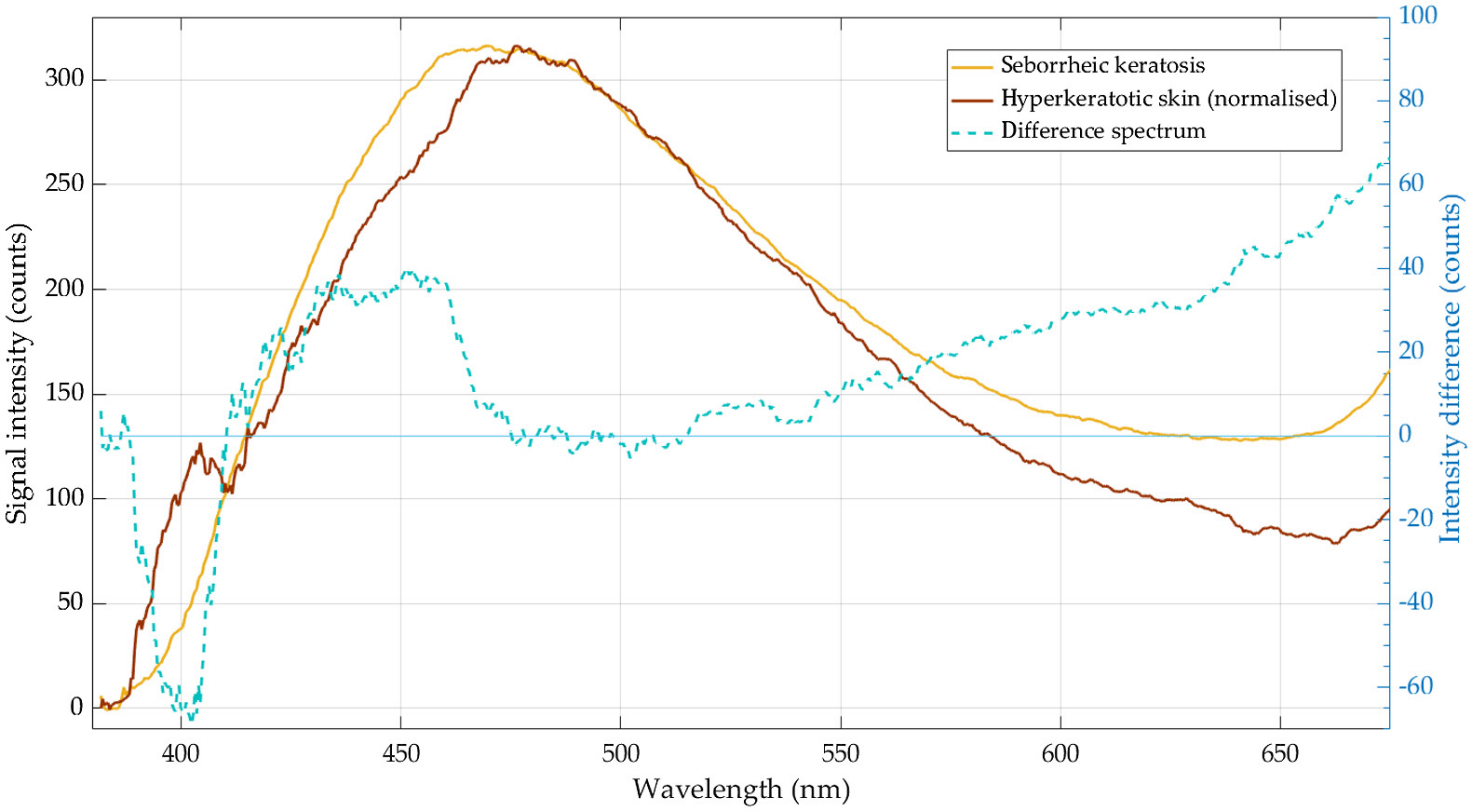
Smoothed spectrum of hyperkeratotic skin (brown line) normalized to the SK maximum intensity and subtracted from the smoothed spectrum of SK (yellow line). The difference spectrum is marked with dashed cyan line.

By subtracting the normalized, smoothed spectrum characteristic for hyperkeratotic skin (Fig. 4, brown line) from the smoothed example spectrum typical for SK (Fig. 4, yellow line), we obtained a difference spectrum (Fig. 4, dashed cyan line) that we used for detailed interpretation of the origin of the measured fluorescence signals.

## 4 Discussion

In dermatofluoroscopy, spectra of PSL are dominated by fluorescence signals resulting from stepwise two-photon excitation of cutaneous fluorophores with 800  nm/1  ns laser pulses. Intensity and spectral shape of cutaneous melanin fluorescence reflect the cancer-induced metabolic alterations in melanocytes^4^^,^^6^^,^^15^ and therefore play an important role in diagnosing CM, the deadliest form of skin cancer. Moreover, fluorescence of other excited fluorophores such as NAD(P)H coenzyme and flavin adenine dinucleotide (FAD) indicates the status of cellular metabolism and therefore serves as important additional information in dermatofluoroscopy. Beside the fluorescence signals, the intensity of the second harmonic of excitation frequency (800 nm) observed at 400 nm is analyzed. A decrease of the second harmonic signal of collagen due to destruction of the collagen cross-link by protease might be a hint on the presence of CM or its invasive growth. In CM, the monotonically increasing signal toward longer wavelengths is dominated by melanin fluorescence, whereas the autofluorescence, i.e., the fluorescence intensity of skin fluorophores other than melanin, is very low. On the contrary, in SKs, the fluorescence intensity of endogenous chromophores other than melanin peaking in the range between 440 and 490 nm is much higher compared to melanoma or healthy skin (Fig. 2), in accordance to research of Borisova^16^ and Lihachev.^17^ Moreover, the SHG at 400 nm is absent or has relatively low intensity.

In the spectral range between 430 and 490 nm, after excitation with two 800 nm photons, the fluorescence of various endogenous skin chromophores could possibly overlap: coenzyme NAD(P)H, keratin, collagen, elastin, or collagen/elastin cross links as well as lipids (lipofuscins and ceroids). Since the dermatofluoroscopic spectrum of SK reveals negligible contribution of SHG, one could conclude that fluorescence excitation is restricted to epidermis or dermal-epidermal junction, most likely due to enlarged epidermis as a result of acanthosis or thickened stratum corneum due to hyperkeratosis. Therefore, the contribution of the structural proteins such as collagen and elastin to the autofluorescence signal of SK in dermatofluoroscopy should be minor. On the other hand, the emission of lipopigments peaks at longer wavelengths, usually around 500 to 600 nm^18^ and thus, the autofluorescence of SKs in dermatofluoroscopy with maximum at around 470 nm should be dominated by the emission of NAD(P)H coenzyme and/or keratin, which is in agreement with extensive studies of Yu et al.^19^ The measurements of normally pigmented thickened hyperkeratotic stratum corneum of the finger and toe (Fitzpatrick skin type 2) revealed the fluorescence spectrum with intensity maximum at around 475 nm. Here the laser is focused in the thickened stratum corneum. Because the principle of dermatofluoroscopy is the two-photon excitation of fluorescence, only molecules within the focus will be photoexcited. Outside of focus, the photon fluxes may not be sufficient for an efficient two-photon excitation of fluorophores with no real electronic state at 800 nm (i.e., all the skin chromophores except melanins). Therefore, for hyperkeratotic skin composed of corneocytes with cytoplasm filled with filamentous keratin, the fluorescence of keratin is expected to dominate the spectrum in the range between 430 and 490 nm. Moreover, the fluorescence intensity of NAD(P)H and flavins should be negligible because of missing proliferating and metabolizing cells.

We therefore conclude that the SK signal in the range between 430 and 490 nm is dominated by keratin fluorescence peaking at 475 nm (in agreement with experiments of keratin from human epidermis by Pena et al.^20^) with some spectrally overlapping contribution of NAD(P)H fluorescence with maximum at around 430 to 460 nm (see difference spectrum in Fig. 4 dashed cyan line). This assignment is in agreement with the measurements of the pure fluorescence spectra of NADH in custom-made solid phantoms and the pure spectra of keratin of human nail, peaking at 450 and 470 nm, respectively (Fig. 3, inset). Foci of complete keratinization (true horn cysts and/or pseudo horn cysts) as well as hyperkeratosis (often associated with the presence of an abnormal quantity of keratin) are characteristic features of SK that may explain high-intensity keratin fluorescence in dermatofluoroscopy compared to other skin lesions such as nevus, melanoma, or basal cell carcinoma.

Similar to other melanocytic lesions, the dermatofluoroscopic signal of SKs in the range between 530 and 650 nm is dominated by pheo- and eumelanin fluorescence (cf., difference spectrum in Fig. 4, dashed line), which exact shape, emission maximum, as well as its relative intensity with respect to other signals allow for a reliable differentiation between malignant and benign pigmented tumors.

In dermatofluoroscopy, the spectrum as shown in Fig. 2 (CM type spectrum, red) is observed in every melanoma measured *in vivo* or *ex vivo*, i.e., lack of a spectrum with these characteristics clearly indicates a benign skin lesion. On the other hand, a “melanoma spectrum” can also be observed in pigmented basal cell carcinoma, in nevi with severe morphological atypia, and, to a much lesser extent in some SKs. An accurate differentiation between these cases and CM by the CE-certified DermaFC medical device is possible with an integrated machine learning algorithm based on SVM. The model analyzes all spectra measured in a PSL of interest (usually several hundred are collected per scan, the number depends on the lesion size) and provides a score that scales linearly with the number of spectra classified as characteristic for melanoma. The SVM classifier was trained, validated, and certified as an inherent part of the DermaFC device in a PMCF study. In the PMCF study, four lesions confirmed by three pathologists as SKs, were correctly diagnosed by the DermaFC as benign with average score equal to 17.8. In this study, 28 PSLs were confirmed by pathology as SK, resulting in more than 5000 dermatofluoroscopic spectra. The average score of SK provided with DermaFC was 14.4.

The spectroscopic profile characteristic for SK (SK type spectrum) was detected for almost all of histopathologically confirmed SKs (27 of 28, Table 2) resulting in very high specificity for the recognition of an important melanoma simulator as benign. The only false-positive SK revealed a relatively high number of spectra characteristic for melanoma as shown in Fig. 2 (red) and only few spectra characteristic for SK. Despite this one misclassification, it is well conceivable, that due to distinctive spectroscopic differences, the SVM algorithm is capable to correctly diagnose SK as a benign skin lesion and to prevent its unnecessary excision.

In this study, five lesions with a suspected clinical diagnosis of SK mimicking melanoma were proven to be benign nevi histopathologically. With dermatofluoroscopy, the measured *in vivo* spectra of four of these five lesions revealed the characteristic spectra of nevi and not of SK. Nevertheless, all five nevi had scores under 28 and thus were correctly diagnosed as benign skin lesions.

Even though we were able to identify a characteristic spectrum to differentiate SK from CM, our study shows some limitations. First, this clinical study did not include all different subtypes of SK. Therefore, our results may not be generalized to other subtypes. Second, it would be desirable to confirm our results in larger future clinical studies. Further studies on rare benign and malignant cutaneous lesions are needed to extent and further improve the diagnostic capacity of dermatofluoroscopy.

## 5 Conclusions

SKs are common benign skin lesions that clinically may resemble nevi or CM. Because SKs are not considered as cancer’s precursors, their excision is superfluous. The dermatofluoroscopy, a new technique for diagnosis of PSLs shows a high sensitivity for *in vivo* detection of melanomas^8^^,^^9^ and pigmented basal cell carcinoma.^21^ In this study, a characteristic spectrum of SK has been identified for the first time with three main fluorophores contributing to the signal: NADH coenzyme with maximum intensity at around 450 nm, keratin peaking at 470 nm, and melanin dominating the signal >550  nm. Consequently, 32 of 33 lesions were correctly classified as benign when using CE-certified DermaFC device with integrated machine learning-based algorithm for data analysis.
